# Autoantibody Discovery, Assay Development and Adoption: Death Valley, the Sea of Survival and Beyond

**DOI:** 10.3389/fimmu.2021.679613

**Published:** 2021-05-27

**Authors:** Marvin J. Fritzler, May Y. Choi, Minoru Satoh, Michael Mahler

**Affiliations:** ^1^ Department of Medicine, Cumming School of Medicine, Calgary, AB, Canada; ^2^ Department of Clinical Nursing, School of Health Sciences, University of Occupational and Environmental Health, Kitakyushu, Japan; ^3^ Research and Development, Inova Diagnostics, San Diego, CA, United States

**Keywords:** autoantibodies, regulatory approval review, diagnostic testing methodologies, immunoassays, orphan autoantibodies

## Abstract

Dating to the discovery of the Lupus Erythematosus (LE) cell in 1948, there has been a dramatic growth in the discovery of unique autoantibodies and their cognate targets, all of which has led to the availability and use of autoantibody testing for a broad spectrum of autoimmune diseases. Most studies of the sensitivity, specificity, commutability, and harmonization of autoantibody testing have focused on widely available, commercially developed and agency-certified autoantibody kits. However, this is only a small part of the spectrum of autoantibody tests that are provided through laboratories world-wide. This manuscript will review the wider spectrum of testing by exploring the innovation pathway that begins with autoantibody discovery followed by assessment of clinical relevance, accuracy, validation, and then consideration of regulatory requirements as an approved diagnostic test. Some tests are offered as “Research Use Only (RUO)”, some as “Laboratory Developed Tests (LDT)”, some enter Health Technology Assessment (HTA) pathways, while others are relegated to a “death valley” of autoantibody discovery and become “orphan” autoantibodies. Those that achieve regulatory approval are further threatened by the business world’s “Darwinian Sea of Survival”. As one example of the trappings of autoantibody progression or failure, it is reported that more than 200 different autoantibodies have been described in systemic lupus erythematosus (SLE), a small handful (~10%) of these have achieved regulatory approval and are widely available as commercial diagnostic kits, while a few others may be available as RUO or LDT assays. However, the vast majority (90%) are orphaned and languish in an autoantibody ‘death valley’. This review proposes that it is important to keep an inventory of these “orphan autoantibodies” in ‘death valley’ because, with the increasing availability of multi-analyte arrays and artificial intelligence (MAAI), some can be rescued to achieve a useful role in clinical diagnostic especially in light of patient stratification and precision medicine.

## Overview

The use of proteomic biomarkers has become a valuable and effective approach to the prediction, diagnosis, and management of individuals with a wide range autoimmune and autoinflammatory diseases (1–3). The spectrum of proteomic biomarkers used in clinical settings includes those with a long history such as C-reactive protein, those associated with the complex pathways involved in the pathogenesis of these diseases, such as anti-dsDNA and anti-citrullinated peptide antibodies (ACPA), interferons and interleukins, which reflect various interactions and responses of inflammatory cells.

To effectively utilize the huge data sets that can now be generated through autoantibody and other biomarker analytics, machine learning and artificial intelligence (AI) in the setting of precision health (PH) are major drivers for biomarker use in clinical practice (2, 4–6). For example, autoantibodies combined with other multi-analyte “omic” profiles are now beginning to form the basis of predicting disease thus allowing for disease prevention strategies and earlier and effective personalized interventions for established disease (7–10). As medical intervention continues to move toward disease prediction and a model of “intent to PREVENT” morbidity and mortality (11), futuristic diagnostics will take into consideration symptoms and risks, as opposed to an established disease and organ involvement approach. Closing the gaps in autoantibody diagnostics will involve newer diagnostic platforms that utilize emerging megatrends such as systems medicine, consumer-driven social networks, AI and deep learning all benefiting a paradigm shift to PH (2).

This manuscript will focus on autoantibodies and the various limitations and gaps that persist in their effective use in clinical practice. To achieve an understanding and appreciation of these limitations, the pathways leading to the discovery and adoption of some autoantibodies and the rejection of others will be explored.

## The Virtuous Cycle of Autoantibody Discovery and Adoption

**Figure 1:**
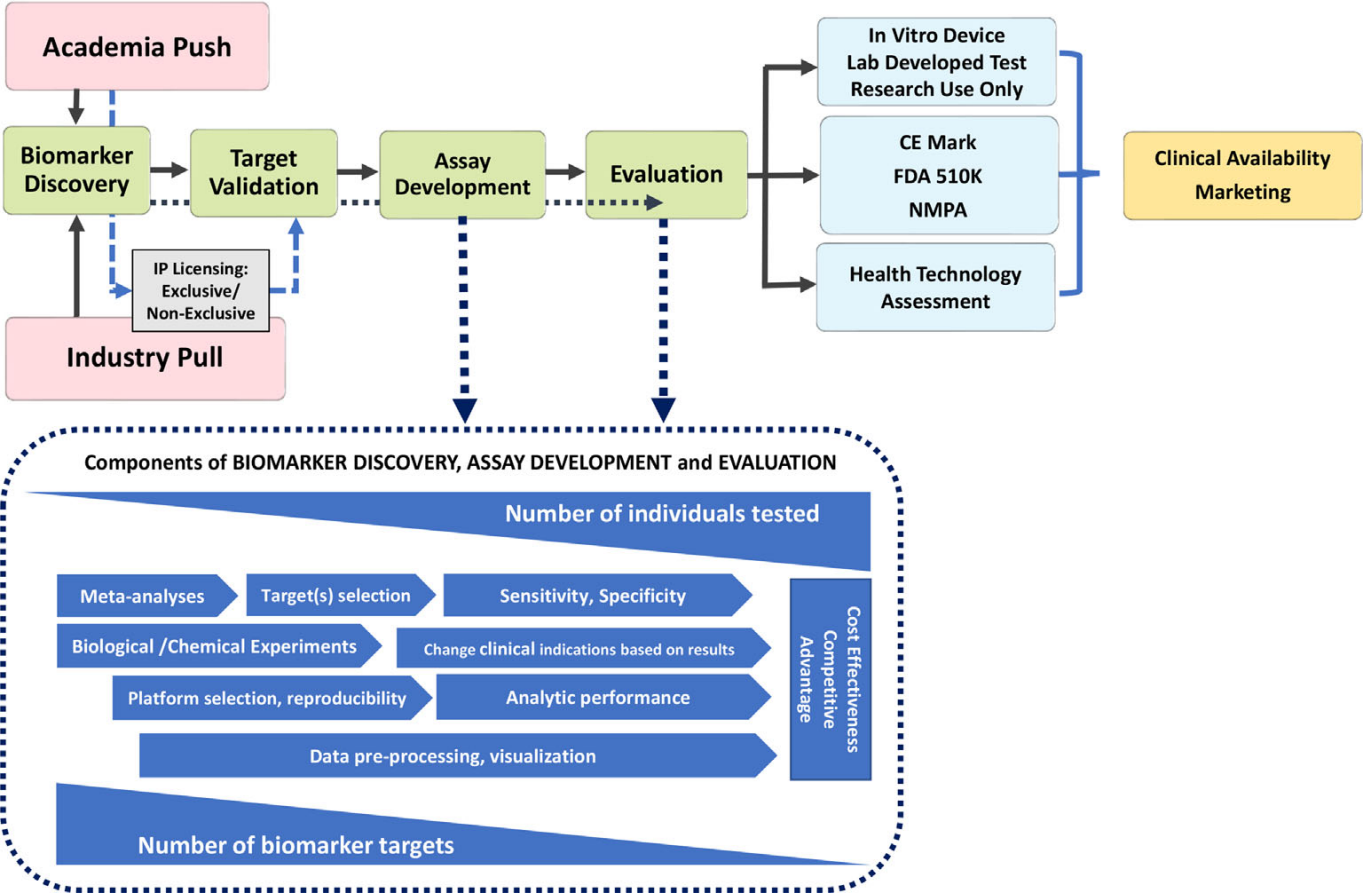
Pathway to Diagnostic Biomarker Translation. Biomarker development process from research and discovery to development and clinical use is a multi-faceted process with a wide range of timelines that undergoes several phases from discovery to clinical availability and utilization. CE marking is a certification mark that indicates conformity with health, safety, and environmental protection standards for products sold within the European Economic Area; the FDA, Food and Drug Administration (USA) and NMPA, National Medical Products Administration in China require, albeit some unique jurisdictional standards and timelines.

To understand why certain autoantibodies are in wide use while others lie dormant or are in very limited use, it is important to review two main overlapping pathways of the “virtuous cycle” of autoantibody innovation (12, 13). The first is the pathway of biomarker discovery and translation (**Figure 1**). Dating to the late 1970s (14), medical sciences witnessed the ‘golden age” of cell and molecular biology, which has in turn served as the hot-bed for autoantibody discovery (15, 16). Historically, autoantibodies were first reported in organ specific autoimmune diseases (17), then in what eventually was called the anti-phospholipid syndrome (18) and in systemic lupus erythematosus (SLE) traced to the discovery of the lupus erythematosus (LE) cell (19). This was followed by a remarkably broad spectrum of autoantibodies in SLE, other systemic autoimmune rheumatic diseases and a growing spectrum of ‘new’ clinical conditions and syndromes, some only regarded as being autoimmune for less than 10 years (20). Again, from a historical perspective, virtually all these autoantibody discoveries were in academic laboratories, but with the realization of a significant market value of autoantibody testing and patented biomarkers, research and development (R&D) divisions of *in-vitro* diagnostic (IVD) companies have also become an important source of these new discoveries.

**Figure 2:**
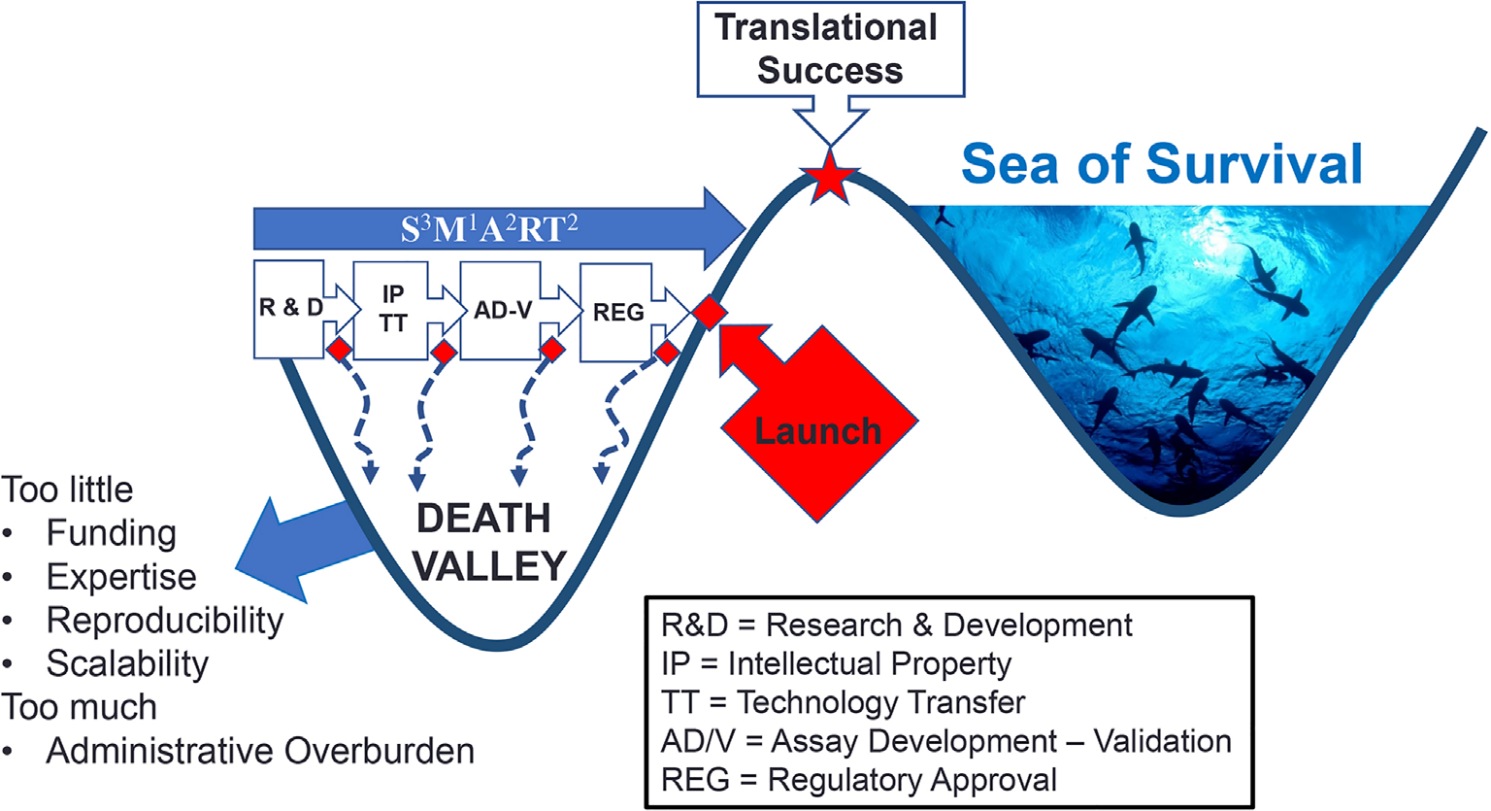
Death Valley of Biomarker Translation. Successful crossing ‘death valley’ is dependent on a number of factors and S^3^M^1^A^2^RT^2^ characteristics (Specific, Sensitive, Scalability, Measurable, Actionable, Added value, Realistic, Titratable, Temporal Timing) leading to variable timelines to Translational Success.

Discovery of a novel autoantibody is only the first very small step on the pathway to adoption in clinical practice (**Figure 1**). While initial claims of diagnostic value (clinical relevance, clinical phenotype, sensitivity and specificity) may be impressive, validation becomes the next critical step to ensure the initial claims are repeatable and followed by exploration in more depth the potential “market value” of the autoantibody (e.g., does it fill a seronegative gap, does it identify an important clinical subset, is it actionable?) Typically, at this stage of autoantibody development, a decision may be taken to patent the novel marker and derive a source of licensing revenues from industry (another onerous process that is not part of this review) and/or be entered into the publication “derby” and achieve the status of primacy (i.e., “first to publish”) and then become open to wider use. A critical step is to determine if the novel autoantibody can be detected by conventional diagnostic platforms [e.g., enzyme linked immunoassay (ELISA), addressable laser bead immunoassay (ALBIA), line immunoassay (LIA), particle-based technology (PMAT) or cell-based assays (CBA)] that are accessible to diagnostic laboratories and thereby achieve wide use. Unfortunately, some novel autoantibody discoveries depend on highly sophisticated techniques and/or protocols that are not thoroughly or clearly described thereby limiting their validation by other investigators and their potential for wide adoption. If the “first to publish” group does not pursue the research on the given biomarker, follow-up studies by other investigators are met with limited access to high impact factor journals because journal editors typically prefer something new and disruptive, and furthermore, granting councils do not see this as innovative, hypothesis-generating research. Obviously, for an autoantibody discovery to successfully find its way through the virtuous pathway of innovation, significant resources and investments are needed from granting councils, R&D budgets, philanthropic donations, and home institutions (universities, colleges, research institutes). In addition, challenges to successful navigation of the pathway come in the form of administrative overburden (“red tape”) to achieve ethical approval, material transfer agreements and intellectual property regulations imposed by academic institutions and funders alike. If a novel autoantibody fails to clear any of these steps, it tends to fall prey of the “valley of death” (21) (**Figure 2**).

Autoantibodies that pass the “acid tests” described above can then proceed to the next phase of optimization wherein issues of assay development like reproducibility, sensitivity, specificity, standardization, clinical applications, cost effectiveness and competitive advantages are rigorously evaluated, typically by a IVD companies (**Figure 1**). Concurrently, thorough evaluation of the realistic market value of the autoantibody in clinical practice is needed because to proceed to IVD regulatory approval [European Union CE mark, Food and Drug Administration USA (FDA) approval, National Medical Products Administration (NMPA) in China, Health Canada, or other regional jurisdictions] requires tremendous paperwork and patience coupled with attention to detail. In some cases, and many times as a temporary measure, while the more rigorous CE and FDA applications are being filed and adjudicated by regulatory agencies, an autoantibody test is offered to clinicians as a “laboratory developed test” (LDT) and with that designation, a disclaimer is required to the same effect. Another approach to bridge the gap between regulatory submission and regulatory approval is to offer the test as a “Research Use Only” (RUO) assay. A limitation of the LDT and RUO approaches is that, in some health care payer systems, reimbursement may not be provided for assays having LDT or RUO status. An intermediate approach is to proceed on a formal Health Technology Assessment (HTA) pathway, which is attended by clearly defined qualifiers and qualifications. Nonetheless, the goal is to achieve the ‘nirvana’ of novel autoantibody innovation and that is full IVD regulatory approval status (e.g., CE mark in the European Union). From there through marketing, the assay is typically widely adopted and with increasing demand by clinicians, is available for clinical use. However, even with the virtuous cycle hurdle having been crossed, the assay enters into a rather competitive, if not hostile, “real world” environment of ‘dog-eat-dog’, competitive edge or what is referred to as the “Darwinian Sea of Survival” (22) (**Figure 2**).

Returning to the ‘death valley’ of innovation, it is important to appreciate a nuance of this metaphor because even in the “real world” of Death Valley (California, USA), while there is widespread evidence of death, there are remarkable evidences of life. Even some rocks, referred to as “wandering”, “sailing” or “walking”, seem to be ‘alive’ (23). This is to remind that although more than 90% of all autoantibodies reported in the literature never achieve IVD regulatory approval (**Table 1**), they should not be regarded as “dead” or having no value. As one example, although greater than 200 different autoantibodies have been described in SLE (24) less than 15 are typically utilized as biomarkers in clinical practice (**Table 1**). While an extensive catalogue of autoantibodies described to date is published (24, 25) a partial list of those that may warrant re-evaluation and rescue of these “orphan autoantibodies” (15, 26–28) from ‘death valley’ is shown in **Table 1**.

**Table 1:** Snapshot of autoantibodies in use (survivors) for systemic autoimmune rheumatic diseases.

Regulatory Status/adoption	SLE	SSc	AIM	RA	SjS	Vasculitis
Widely available	dsDNA	CENP-A, -B	Jo-1 U1-RNP	RF	SS-B/La	PR-3
	Sm	TopoI/Scl-70	HMGCR	ACPA	SSA/Ro60	MPO
	U1-RNP	RNA Pol III	Ro52/TRIM21		Ro52/TRIM21	
	Chromatin	Ro52/TRIM21				
	Histone	U1-RNP				
	SS-A/Ro60					
	SS-B/La Ro52/TRIM21					
	APLA					
Geographic dependent	Ribosomal P	Th/To	ARS	Ra33	Alpha fodrin	Elastase
	PCNA	Fibrillarin	SRP		DFS70*	BPI
	Ku	PM/Scl	MDA-5			Lactoferrin
	DFS70*	NOR-90	SAE			DFS70*
		DFS70*	TIF1y/155			
			NXP-2			
			Mi-2			
			PM/Scl			
			Ku			
			DFS70*			
Primarily RUO/LDT	NMDAR2	RuvBL1/2	NT5c1A/Mup44	CarP	NuMA	Lysozyme
		BICD2				Azurocidin
		U11/U12 (RNPC-3)				
		Exosome				
		eIF2B				

*aid in the exclusion of diagnosis.

ACPA, anti-citrullinated peptide antibodies; AIM, autoimmune inflammatory myopathies; APLA, anti-phospholipid antibodies; ARS, aminoacyl tRNA synthetase; BICD2, bicaudal D2; BPI, bacterial permeability inhibitor; CarP, carbamylated protein, CENP, centromere protein; DFS, dense fine speckled; dsDNA, double-stranded deoxyribonucleic acid; eIF2B, guanine nucleotide exchange factor; HMGCR, 3-hydroxy-3-methyl-glutaryl-coenzyme A reductase; Ku, named after the index patient serum (Kuriowa), a dimeric 70-/80-kDA protein complex that binds to DNA double-strand break ends and is required for DNA repair; LDT, laboratory developed test; MDA, melanoma differentiation-associated protein 5; MPO, myeloperoxidase; NMDAR, N-methyl-D-aspartate receptor; NOR, nucleous organizer protein; NuMA, nuclear mitotic apparatus; NT5c1A, Nucleotidase 5' Cytosolic IA; NXP, nuclear matrix protein 2; PAD, protein/peptidyl arginine deiminase; PCNA, proliferating cell nuclear antigen; PDGFR, platelet derived growth factor; PR3, proteinase 3; RF, rheumatoid factor; RNP, ribonucleoprotein; RUO, research use only; SRP, signal recognition particle; TIF, transcription intermediary factor; TopoI, topoisomerase I; TRIM, tripartite motif.

## Rescuing Autoantibodies From Death Valley

While some autoantibodies appear to have perished in ‘death valley’ (**Table 2**), there are a number of reasons to “rescue” them. With the advent of multi-analyte arrays with algorithmic analysis (MAAA) as an approach to PH (2, 3, 66) the value of these autoantibodies may be discovered when they are combined and permutated with other biomarkers, and hence fill seronegative gaps such as in antinuclear antibody (ANA)-negative SLE (67, 68) and other systemic autoimmune rheumatic diseases (SARD) (69–73). In addition, machine learning and AI approaches may find that these autoantibodies provide value in determining subsets of disease that have a more clinically actionable basis (3). In addition, on future exploration, ‘death valley’ autoantibodies may have value predicting the evolution of very early SARD (i.e., undifferentiated connective tissue disease, UCTD) to confirmed, criteria-defined SARD. For example, 5-50% of UCTD patients or very early connective tissue disease evolve to fulfill diagnostic and classification criteria of a SARD. Of the UCTD patients that do evolve to a SARD, the majority (80%) have been reported to develop SLE, while of the reminder, some evolve to systemic sclerosis (SSc), Sjögren syndrome (SjS), rheumatoid arthritis (RA) and autoimmune inflammatory myopathies (AIM) (74–78). Some predictive autoantibodies and their temporal appearance, especially in RA, are known but longitudinal studies of very early SARD/UCTD are required and it is here that ‘death valley’ autoantibodies may find important predator/prognostic value. Recognizing this, there has been a call for more studies to identify diagnostic, prognostic (i.e., disease activity, remission, and outcomes) and biomarkers that predict earlier autoimmune disease onset, as well as biomarkers that predict effectiveness of a growing spectrum of therapeutic options [reviewed in (5, 28, 79)].

**Table 2:** Death Valley Autoantibodies of Interest that might address ‘Seronegative Gaps’ in Systemic Autoimmune Rheumatic Diseases.

SARD	% of patients with no identifiable autoantibody*	Death Valley Autoantibodies and Comments
**SLE**	**3-25**	ASE1 anti-sense ERCC1 (nucleolar SLE) (29); cell cycle SG2NA associated with cancer (30–32); replication protein A (RPA) complex (33), RNA helicase A, Ki/SL (33), Ago2 (34)
**SSc**	**3-10**	Bicaudal D2 (BICD2) (35); U11/U12 (RNPC-3) (36, 37); HMGs (38); B23/nucleophosmin (39), eIF2B (40)
**RA**	**15-50**	Newer biomarkers such as antibodies to PAD1, PAD2, PAD3, and PAD4, as well as to carbamylated peptides/proteins are narrowing the seronegative gap (9, 41)
**AIM**	**20-30**	Survival of motor neuron (SMN) complex (42, 43); PUF60 (44–46); NT5c1A/Mup44 not just associated with sIBM (47–49)
**SjS/Sicca syndrome**	**10-30**	CENP-C (50–52), α-fodrin (53, 54), NA14/SSN1 (55); SS56 (56); golgins (57), TS-1 RNA; M3 receptor (58, 59), Ki/SL (33)
**Vasculitis**	**10-30**	NA14 and CK15 (60); LAMP2 (61); EEA1 (62)
**Other**	–	p80/coilin: associated with DFS70 (63), Ge-1/GW182 (57, 64)

AIM, autoimmune inflammatory myopathies; ANA, anti-nuclear antibody; CENP, centromere protein; HMG, high mobility group proteins; NA, nuclear antigen; PAD, protein arginine deiminase; RA, rheumatoid arthritis; SG2NA, S- G2-phase nuclear antigen; sIBM, sporadic inclusion body myositis; SjS, Sjögren syndrome; SLE, systemic lupus erythematosus; SSc, systemic sclerosis.

*Wide ranges due to demographic and clinical variability of cohorts. In recent SLE classification criteria (65), ANA is a required criterion, hence the percent ‘seronegative’ is, by definition, zero. However, having a positive ANA does not necessarily mean a relevant disease autoantibody will be detected.

In addition to providing more information as predictors of SARD, it is plausible that death valley of autoantibodies also hold important value for other key functions of autoantibodies such as their pathogenic (80, 81), protective (26) and prognostic values (27, 82). For example, despite substantial advances, the high morbidity and mortality that currently characterizes SLE can largely be attributed to a delay in diagnosis, gaps in our understanding of the role of autoantibodies in early disease, and limited effective therapeutic options. SSc is another SARD with heterogeneous clinical features that is extremely difficult to diagnose in the early phase (83), resulting in a critical delay in therapy which is often begun when internal organ involvement is already irreversible (77, 78). Older classification criteria (84–86) had a remarkably low sensitivity for the early phase of disease (87) so they were replaced by the American College of Rheumatology (ACR)/European League Against Rheumatism 2013 criteria which improved the disease classification (88). Nevertheless, the diagnosis may be delayed for several years after the onset of Raynaud’s phenomenon (RP) or certainly after the first non-RP symptom. RP, ANA positivity, and puffy fingers were recently indicated as “red flags” (by the Very Early Diagnosis Of Systemic Sclerosis (VEDOSS) study)–that is, the main elements for suspicion of SSc in the very early phase of the disease (89). Confirming the diagnosis requires further tests, particularly nailfold videocapillaroscopy and evaluation of disease specific autoantibodies (**Table 1**). In this way, patients can be identified in the very early phase of disease enabling a “window of opportunity” whereby the physician can act with effective drugs to block or at least slow the progression of the disease (10, 81, 90–92). The principal challenge is to detect valid predictors of disease evolution to enable treatment of patients in the early stage of disease. Perhaps lying in ‘death valley’ are the key autoantibodies that can facilitate these goals.

An early and accurate diagnosis of SLE and other SARD through the use of autoantibody testing that has met SSSMAART(specificity, sensitivity, scalability, measurable, actionable, added value, realistic, titres, timely) characteristics (3) (**Figure 2**) will help improve SARD-associated clinical outcomes and healthcare expenditures. Clearly, not all ‘death valley’ autoantibodies should be expected to provide value because there is compelling evidence that the vast majority of autoantibodies studied to date are “indifferent” (93) or “junk” autoantibodies (15). However, as a word of caution, it should be recalled that shortly after the completion of the human genome project it was assumed that a significant portion of the human genome was “junk”, only to discover unanticipated functions of DNA were yet to be discovered (94, 95). Accordingly, we prefer the term ‘orphan” autoantibodies over “junk” autoantibodies to categorize those which have no known or proven function (2, 15, 26).

As briefly outlined above, it is well-established that there is an increasing use, awareness and focus on PH and disease prevention (2). PH applied to SARD will require paradigm shifts in the use and application of autoantibodies and other biomarkers. For example, autoantibodies combined with other multi-analyte “omic” profiles will form the basis of disease prediction allowing for earlier intervention linked to disease prevention strategies, as well as earlier, effective and personalized interventions for established disease (2, 5). As medical intervention moves to disease prediction and a model of “intent to PREVENT,” diagnostics will include an early symptom/risk-based, as opposed to a disease-based approach. Newer diagnostic platforms that utilize emerging megatrends such as AI and close the gaps in autoantibody diagnostics will benefit from paradigm shifts thereby facilitating the PH agenda.

## Technology and Cooperation to the Rescue

Single autoantibody testing only provides a narrow window of the clinical picture and also does not represent the ultimate approach to an early and accurate diagnosis or following or predicting responses to therapeutic interventions. Accordingly, multi-analyte techniques for detecting multiple autoantibodies on MAAA are coming into use (28, 96). With the advent of MAAA, emerging evidence indicates that when certain combinations of biomarkers, such as the interferon signature and stem cell factor accompany autoantibody and ANA results, the predictive power for SLE is markedly increased (28). A few examples of MAAA that have emerged include the SLE-Key rule out test, (97, 98) that uses microarray technology to identify autoantibody patterns that discriminate SLE from healthy: reported 94% sensitivity; 75% specificity; 93% negative predictive value. The Avise^®^ Lupus Test uses a parallel approach to detect autoantibodies and cell-bound complement products to distinguish SLE from other rheumatological conditions (99) and may predict disease progression in patients who had non-specific clinical signs (100). However, the relatively low sensitivity, suggests that patients with preclinical SLE could go undetected (101). And last, the VectraDA blood test, based on measuring the concentrations of 12 biomarkers that reflect the pathogenesis of RA, is designed to provide an objective measure of disease activity for RA by providing a score on a scale of 1 to 100 with high scores associated with disease progression (102). The analytical validity, clinical validity, and clinical utility of VectraDA have been reported and it is reputed to assist in monitoring clinical responses to disease-modifying anti-rheumatic drugs (102), including blockade of the CD40/CD40L pathway (103). Based on a wide range of clinical studies on VectraDA, the ACR has recently added Vectra DA as one of the methods to assess disease activity. It has also been shown to have value in following the clinical progression and remission of Adult Onset Still’s disease (104).

## Avoiding Death Valley and Surviving the ‘Darwinian Sea of Survival’

It is important to consider ways in which autoantibody discovery can result in a more rapid and protective transition to an actionable biomarker with proven clinical value and availability in mainstream diagnostic testing. First, it is important to appreciate the “push” and “pull” equation of innovation (**Figure 1**). While autoantibody discovery continues by academics, supported by largely institutional investors (i.e., granting councils), this is only the “push” side of innovation. For an autoantibody, or any biomarker, to succeed it needs to meet a need or a demand (i.e., fill a seronegative gap; identify a disease subset with a specific actionable therapeutic choice) and, hence, have a strong “pull” component from the diagnostic industry, regulators, physicians, laboratory scientists, patient advocates, health care payers, and angel investors. Without a well-balanced push-pull ‘equation’, it is unlikely that an autoantibody will make it across the ‘death valley’ of innovation.

Second, in the discovery research phase autoantibodies must be shown to be S^3^M^1^A^2^RT^2^ (**Figure 2**): demonstrate **S**pecificity, **S**ensitivity and **S**calability, **M**easurable using conventional technologies, **A**dd value to clinical management, be **A**ctionable (lead to or suggest a clinical decision), **R**ealistic (detection should not involve complicated processes or procedures) and address the **T**emporal **T**iming during the course of the disease (i.e. is it predictive or transient) (3). The latter is an important factor because not all autoantibodies are present at diagnosis and some do not persist throughout the disease course (105). Early attention to these factors can help assure that the autoantibody will not only survive ‘death valley’ but also the ‘Darwinian Sea of Survival’ (**Figure 2**).

The 'Darwinian Sea of Survival' in deference to Death Valley was a metaphor initially intended to describe the entire span of innovation (3) including the way it is used here as the end stage struggle for survival in a highly competitive market where constantly changing medical advances, technologies, clientele needs and expectations, and investment strategies are constantly being evaluated. Despite an initial phase of triumph of having successfully traversed ‘death valley’, some innovations simply do not survive in the ‘sea of survival’ because of technological advances, economic considerations (investors, managers, client’s shifting priorities) and an increasing trend to central procurement where other factors that do not include true performance of a biomarker may not be the primary factor of interest.

## Diagnostic Industry Challenges

From the industry perspective, increased regulatory burden especially due to in-vitro device regulation (IVDR) is making the rescue of autoantibodies from the ‘death valley’ very challenging. This new regulation requires additional evidence about the usefulness of the biomarker beyond the clinical validity. Clinical utility studies and likely health economic studies will be especially required for novel biomarker rescue and approval. In addition, the diagnostic platforms available at a given IVD company can also have a significant impact on the success of a biomarker. While some autoantibodies are useful as a standalone marker, other autoantibodies require a panel of markers to be tested at the same time. A typical example for biomarkers that should be measured as a multi-analyte panel are myositis-specific antibodies (106).

Quality control, both during the manufacturing process, and the clinical setting, requires the availability of patient samples that can serve as calibrators of controls (106). While this is achievable for common markers (such as ACPA), it can represent a significant challenge for orphan autoantibodies (e.g., U11/U12 RNP, RNPC-3). Human or humanized recombinant antibodies represent a viable, but not yet cost-intensive alternative.

Although AI might provide new approaches to combine autoantibody results in scores that provide increased clinical value (107), this opens additional challenges from the regulatory perspective. Along those lines, the FDA just published an action plan to outline activities and areas of focus to manage software as medical devices (SaMD) that leverage AI [reviewed in (108)].

## Summary

The discovery of novel autoantibodies is linked to an ever-expanding spectrum of autoimmune conditions. For a number of reasons, the vast majority of discovered autoantibodies are not currently used in routine clinical diagnostics and have become relegated to the ‘death valley’ of innovation. With the advent of PH and MAAA it seems plausible that some of these autoantibodies might be ‘rediscovered’ and become valuable predictive, prognostic and actionable biomarkers. In the meantime, successful innovation is a ‘real time’ partnership with a balance of the push and pull forces of innovation.

## Patient-Public Participation

Patients or the public were not involved in the design, or conduct, or reporting, or dissemination plans of our research.

## Author Contributions

MF and MC conceived of the content. MF, MC, MS, and MM conducted the literature review and wrote and/or edited manuscript drafts. MC, MF, and MM provided graphical content. All authors contributed to the article and approved the submitted version.

## Conflict of Interest

MM is an employee of Inova Diagnostics. MF is the Director and MC is the Associate Director of MitogenDx. MF is a consultant for and received speaking honoraria from Inova Diagnostics Inc (San Diego, CA) and Werfen International (Barcelona, Spain).

The remaining authors declare that the research was conducted in the absence of any commercial or financial relationships that could be construed as a potential conflict of interest.

The handling editor declared a past collaboration with one of the authors MF.

## Abbreviations

ACPA, anti-citrullinated peptide antibodies; AI, artificial intelligence; AIM, autoimmune inflammatory myopathies; ANA, anti-nuclear antibody; APLA, anti-phospholipid antibodies; BICD2, bicaudal D2; BPI, bacterial permeability inhibitor; CENP, centromere protein; DFS, dense fine speckled; dsDNA, double-stranded deoxyribonucleic acid; FDA, Food and Drug Administration (USA); HMGCR, 3-hydroxy-3-methyl-glutaryl-coenzyme A reductase; IVD, *in vitro* diagnostic; Ku, named after the index patient serum (Kuriowa), a dimeric 70-/80-kDA protein complex that binds to DNA double-strand break ends and is required for DNA repair; LDT, laboratory developed test; MAAAA, multi-analyte arrays with analytic algorithms; MDA, melanoma differentiation-associated protein 5; MPO, myeloperoxidase; NMDAR, *N*-methyl-D-aspartate receptor; NOR, nucleous organizer protein; NMPA, National Medical Products Administration; NuMA, nuclear mitotic apparatus; NXP, nuclear matrix protein 2; PAD, protein arginine deiminase; PCNA, proliferating cell nuclear antigen; PDGFR, platelet derived growth factor; PH, precision health; PR3, proteinase 3; RA, rheumatoid arthritis; RF, rheumatoid factor; RNP, ribonucleoprotein; RUO, research use only; SaMD, software as medical device; SGNA, S- G-phase nuclear antigen; sIBM, sporadic inclusion body myositis; SjS, Sjogren syndrome; SLE, systemic lupus erythematosus; SRP, signal recognition particle; SSc, systemic sclerosis; TIF, transcription intermediary factor; TopoI, topoisomerase I; TRIM, tripartite motif.
